# Supplementary material for: From Hue to Health: Exploring the Therapeutic Potential of Plant-Pigment-Enriched Extracts
Source: Microorganisms. 2025 Aug 4;13(8):1818. doi: 10.3390/microorganisms13081818 (PMC12388485; doi:10.3390/microorganisms13081818)
Supplement: Supplementary file 1 [file microorganisms-13-01818-s001.zip › microorganisms-3746235-supplementary.pdf]

Table S1. Quantification of Pigment Concentrations (Chlorophyll, Carotenoids, Anthocyanins) in Various Plants.

Plant	Chlorophyll content			Plant	Carotenoid content (µg/100g)	Plant	Anthocyanin content (µg/100 g)
	Chlorophyll a (µg/g)	Chlorophyll b (µg/g)	All chlorophyll (µg/g)				
**Moringa oleifera**	20.48	7.89	28.37	***Curcuma longa*** **(Turmeric)**	180.8	***Beta vulgaris*** **(Beetroots)**	30.76
**Nerium oleander**	19.11	9.21	28.31	***Capsicum annuum*** **(Pepper)**	159	***Hibiscus sabdariffa*** **(Roselle petals)**	5.06
Olea europaea **(Olives)**	12.60	5.21	17.81	***Daucus carota*** **(Carrot)**	120.5	***Solanum melongena*** **(Eggplant peels)**	10.39
Ocimum basilicum **(Basil)**	7.85	3.28	11.13	***Solanum lycopersicum*** **(Tomato)**	173.6	***Brassica oleracea*** **var.** ***capitata*** **f.** ***rubra*** **(Red cabbage)**	14.91
Citrus limon **(Lemon)**	18.77	8.63	27.40			***Prunus domestica*** **(Plum)**	1.14

"All chlorophyll" refers to the sum of Chlorophyll a (Chl a) and Chlorophyll b (Chl b).

The present study's findings on pigment content in various plants can be contextualized by comparing them with existing research. [1] reported significantly higher total chlorophyll concentrations (59.6-82.6 µg/mL) in medicinal plants compared to our results (11.13-28.37 µg/g). This discrepancy may stem from differences in plant species, extraction methods, or the units of measurement (µg/mL vs. µg/g). Their observation of Mimosa pudica exhibiting the highest chlorophyll aligns with the potential for high photosynthetic activity in certain medicinal plants. In terms of carotenoids, Jeyanthi Rebecca et al., (2014) found concentrations ranging from 1.3 to 18.3 mg/100g in vegetables, with Daucus carota (carrot) showing the highest levels. While our study reported carotenoid content in µg/100g, direct comparison reveals that Curcuma longa in our study displayed a very high concentration of carotenoids. This highlights the importance of considering the specific plant part used and the potential for variations in pigment accumulation across different species. Regarding anthocyanins, Ali Shehat et al., (2020) reported higher concentrations in plum peels (60.45 mg/100g) compared to our study's plum content (1.14 mg/100g). This difference could be attributed to variations in plum varieties, ripeness, or extraction techniques. Furthermore, their results show that fruit peels tend to contain much higher concentrations of anthocyanins than the flesh, this is a very important consideration. Collectively, these comparisons underscore the influence of species, plant part, growth conditions, and extraction methods on pigment quantification, emphasizing the need for standardized protocols and careful interpretation of results.

Table S2. Effect of chlorophyll pigment from different plants on the growth of pathogenic organisms.

**Microbial isolates**	**Chlorophyll pigment Diameter of the inhibition Zone(cm)**					***p*-value**
	**Moringa (*Moringa oleifera)***	**Nerium (*Nerium oleander*)**	**olives (*Olea europaea*)**	**Basil (*Ocimum basilicum*)**	**Lemon (*Citrus limon*)**	
S. aureus	0.0	0.0	1.5±0.0	0.0	0.0	<.0001
Klebsiella pneumoniae	0.0	0.0	1.3±0.0	0.0	0.0	<.0001
Klebsiella oxytoca	0.0	0.0	2±0.0	0.0	0.0	<.0001
Pseudomonas fluorescens	0.0	0.0	0.0	0.0	0.0	NE
Pseudomonas aeruginosa	0.0	0.0	2±0.0	0.0	0.0	<.0001
E. coli	0.0	0.0	1.5±0.0	0.0	0.0	<.0001
Salmonella typhimurium	0.0	0.0	1.3±0.0	0.0	0.0	<.0001
Pasteurella haemolytica	0.0	0.0	1.3±0.0	0.0	0.0	<.0001
Morganella morganii	0.0	0.0	1.6±0.0	0.0	0.0	<.0001
Shigella flexneri	0.0	0.0	2.0±0.0	0.0	0.0	<.0001
Candida albicans	0.0	0.0	0.0	0.0	0.0	NE
Aspergillus flavus	0.0	0.9±0.0	1.7±0.0	1.06±0.20	0.0	<.0001
Aspergillus niger	0.0	1.6±0.0	0.0	2.03±0.15	0.0	<.0001

NE, non-estimate.

Table S3. Effect of carotenoid pigment from different plants on the growth of pathogenic organisms.

**Microbial isolates**	**Carotenoid pigment Diameter of the inhibition Zone(cm)**				***p*-value**
	**Tumaric (*Curcuma longa*)**	**Pepper (*capsium annuum*)**	**Carrot (*Daucus carota*)**	**Tomato (*Lycopersicon esculentum*)**	
S. aureus	0.0	0.0	1.13±0.05	0.0	<.0001
Klebsiella pneumoniae	0.0	1.63±0.20	0.0	1.53±0.53	<.0001
Klebsiella oxytoca	0.0	0.0	0.0	0.0	NE
Pseudomonas fluorescens	0.0	0.0	0.0	1.2±0.0	<.0001
Pseudomonas aeruginosa	0.0	0.0	0.0	0.0	NE
E. coli	0.0	0.0	0.0	0.0	NE
Salmonella typhimurium	0.0	0.0	0.0	0.0	NE
Pasteurella haemolytica	0.0	0.0	0.0	0.0	NE
Morganella morganii	0.0	2.03±0.15	2±0.0	0.0	<.0001
Shigella flexneri	0.0	1.86±0.41	0.0	0.0	<.0001
Candida albicans	0.0	0.9±0.0	1.7±0.0	1.06±0.20	<.0001
Aspergillus flavus	2.1±0.17	1.23±0.15	2.16±0.25	2.03±0.17	0.0010
Aspergillus niger	1.96±0.05	1.83±0.05	1.43±0.40	1.86±0.05	0.0649

NE, non-estimated.

The use of herbal extracts as adjuvants in the treatment of microbial infections has garnered considerable research interest. Numerous plant extracts and secondary metabolites possess diverse biological activities, including notable antimicrobial properties [4].

Ahmadi et al., (2022) investigated the effects of varying chlorophyll concentrations on several pathogenic microorganisms. Their findings, using both agar disk diffusion and microdilution assays, revealed a general trend in bacterial resistance: *Listeria* < *Staphylococcus* < *Salmonella* < *Escherichia* < *Pseudomonas*. Specifically, in the microdilution assay, they observed *Listeria* < (*Staphylococcus* = *Escherichia* = *Salmonella*) < *Pseudomonas*. Furthermore, they reported significant differences in the inhibitory effects of different total chlorophyll concentrations against *Listeria* and *Staphylococcus* ($p < 0.05$).

Table S4. Comprehensive List of Compounds Identified in *Hibiscus sabdariffa* Extract by GC-MS Analysis

RT (min)	Compound Name(s) (Tentative Identification)	Area %	Molecular Formula	Molecular weight
5.20	2-Pentadecyl-4,4,7,7-tetradeutero-1,3-dioxapane; Dimethyldiphenyltethylidylpyrrolidine; 2-Methylmalonic acid; Methyl-9,9,10,10-d4-octadecanoate; 1-Felinine	19.09	C20H36D4O2	316
			C20H23N	277
			C4H6O4	118
			C19H34D4O2	302
			C8H17NO3S	207
5.75	7-Nonenoic-7,8-d2 acid, methyl ester; **1-Deoxy-d-arabitol**; 1,3-Pentanediol, 4-methyl-2-nitro-;	12.45	C10H16D2O2	316
			C7H14O5	178
			C6H13NO4	163
			C6H13NO4	163
			C6H14O5	166
6.60	Benzene, 1,3,5-trimethyl-; 1-Isopropenyltricyclo[3.1.0.0(2,6)]hexane; Benzene, 1-ethyl-4-methyl-; Benzene, 1,2,3-trimethyl-	3.18	C9H12	120
6.80	Nodularin; 1H-Indene-1-carboxaldehyde, 2,3,3a,4,7,7a-hexahydro-2-methoxy-, (1à,2á, 3Aà, 7Aà)-; 2,5-Methylene-d,l-rhamnitol; 1,1-Cyclobutanedicarboxamide, 2-phenyl-N,N'-bis(1-phenylethyl)-	2.03	C41H60N8O10	824
			C11H16O2	180
			C7H14O5	178
			C28H30N2O2	426
7.10	Benzenepentanenitrile, α-butyl-δ-oxo-; 7,7-Dimethyl-tetracyclo[4.1.0.0(2,4).0(3,3)]heptane; Benzenepentanenitrile, α-methyl-δ-oxo-; 7,7-Dimethylnorbornadiene; Benzene, 1,3,5-trimethyl-	8.62	C15H19NO	229
			C9H12	120
			C12H13NO	187
			C9H12	120
7.71	Propanedioic acid, 2-(3-hydroxypropyl)-; Glucopyranose, 1,5-anhydro-1-C-tetradecyl-; 1,4-Anhydro-alpha-D-galactopyranose; 2-Hydroxy-2-(4-methoxyphenyl)acetic acid	2.92	C6H10O5	146
			C20H40O5	360
			C6H10O5	162
			C9H10O	182

8.30	1,2,3-Propanetriol, 1-acetate; Ethylene glycol, 2-ethylhexyl ether; Ethanol, 2-(2-ethoxyethoxy)-	0.76	$C_5H_{10}O_4$	134
			$C_{10}H_{22}O_2$	174
			$C_6H_{14}O_3$	134
8.46	**D-Glucose, 6-O-α-D-galactopyranosyl-**; 2-Deoxy-D-galactose; 1,5-Anhydro-D-glucitol, 6-deoxy-3-O-α-D-glucopyranosyl-	1.54	$C_{12}H_{22}O_{11}$	342
			$C_6H_{12}O_5$	164
			$C_{12}H_{22}O_{10}$	326
9.32	**2-Furancarboxaldehyde, 5-(hydroxymethyl)-**; D-Ribose; Tetrahydro-2-furoic acid	1.77	$C_6H_6O_3$	126
			$C_5H_{10}O_5$	150
			$C_5H_8O_3$	116
9.92	**2,3-Dihydroxypropyl stearate**; 3-Pyridinecarboxamide, 2-chloro-N,N-diethyl-; Heptanedioic acid, 4-ethyl-	0.98	$C_{21}H_{42}O_4$	358
			$C_{10}H_{13}ClN_2O$	212
			$C_9H_{16}O_4$	188
11.03	**1,6-Anhydro-β-D-glucopyranose**; 1,6-Anhydro-β-D-mannopyranose; 2,3-Dihydroxypropyl octadecanoate; 2-Ethyl-1-hexanol	2.69	$C_6H_{10}O_5$	162
			$C_6H_{10}O_5$	162
			$C_{21}H_{42}O_4$	358
			$C_8H_{18}O$	130
29.58	**Oleic Acid**; 9-Octadecenoic acid (Z)-; Hexadecanoic acid, ethyl ester	1.35	$C_{18}H_{34}O_2$	282
			$C_{18}H_{36}O_2$	284
32.85	1-Hexadecanol; **9,12-Octadecadienoic acid (Z,Z)-**; Octadecanoic acid	3.92	$C_{16}H_{34}O$	242
			$C_{18}H_{32}O_2$	280
			$C_{18}H_{36}O_2$	284
34.67	Octadecanoic acid, ethyl ester; Phthalic acid, di(2-ethylhexyl) ester; 1-Octadecanol	1.39	$C_{20}H_{40}O_2$	312
			$C_{24}H_{38}O_4$	390
			$C_{18}H_{38}O$	270
34.74	**Stigmasterol**; **α-Tocopherol**; Campesterol	2.29	$C_{29}H_{48}O$	412

			C29H50O2	430
			C28H48O	400
36.60	**α-Tocopherol**; **β-Sitosterol**; Stigmasterol	1.97	C29H50O2	430
			C29H50O	414
			C29H48O	412
43.59	Ethyl iso-allocholate; 4H-1-Benzopyran-4-one, 2-(3,4-dihydroxyphenyl)-6,8-di-α-D-glucopyranosyl-5,7-dihydroxy-; 2,6,10,14,18,22-Tetracosahexaene, 2,6,10,15,19,23-hexamethyl-	3.80	C26H44O5	436
			C27H30O16	610
			C30H50	410
43.71	Docosane, 1-bromo-; 9,12,15-Octadecatrienoic acid, (Z,Z,Z)-; Ethyl linolenate	0.50	C22H45Br	390
			C18H30O2	278
			C20H34O2	306
44.14	Vitamin E; 3,7,11,15-Tetramethyl-2-hexadecen-1-ol; **Phytol**	3.70	C29H50O2	430
			C20H40O	296
44.24	**β-Sitosterol**; Cholesterol; Stigmasterol	4.46	C29H50O	414
			C27H46O	386
			C29H48O	412
44.48	**α-Tocopherol**; Campesterol; **β-Sitosterol**	4.88	C29H50O2	430
			C28H48O	400
			C29H50O	414
44.78	**2-(3,4-Dimethoxyphenyl)-3,5-dihydroxy-7-methoxyflavone**; Flavone 4'-OH,5-OH,7-di-O-glucoside	0.59	C18H16O7	344
			C27H30O15	594
44.94	Ethyl iso-allocholate; 4H-1-Benzopyran-4-one, 2-(3,4-dihydroxyphenyl)-6,8-di-α-D-glucopyranosyl-5,7-dihydroxy-; 4H-1-Benzopyran-4-one, 2-(3,4-dimethoxyphenyl)-...	1.25	C26H44O5	436
			C27H30O16	610
45.07	2H-1-Benzopyran-2-one, 7-(α-L-arabinofuranosyloxy)-; 7-Methoxy-2H-1-benzopyran-2-one; 2-Hydroxy-3,3',4',5',7-pentamethoxyflavone	0.36	C14H14O8	310

$C10H8O3$	176
$C20H20O8$	388

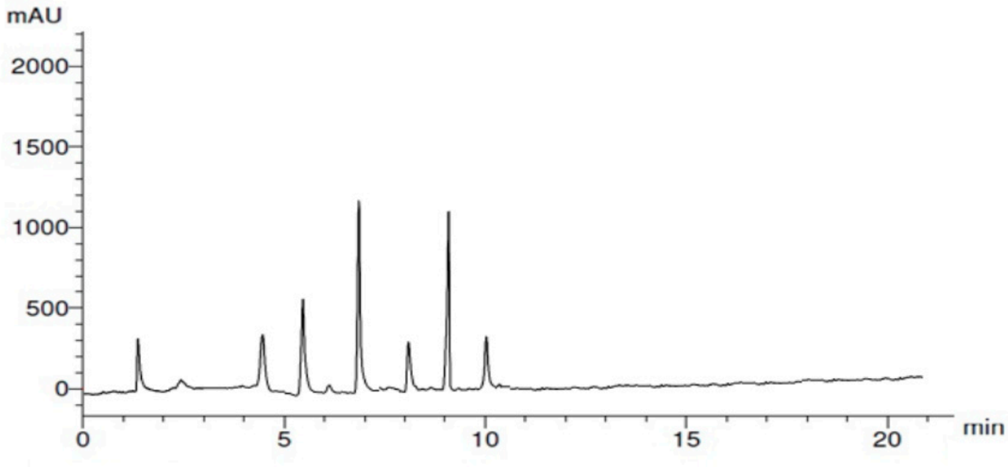

Figure S1. HPLC Chromatogram and Quantification of Major Anthocyanin Glycosides Identified in Hibiscus Pigment.

Refrences

1. Soni, D.K.; Shahi, S.K.; Khandel, P.; Mahobiya, D.; Singh, R.; Yadaw, R.K.; Kanwar, L. Extraction and Estimation of Chlorophylls from Epiphytic Orchids and Their Antioxidants Scavenging Activityanalysis. *Plant Arch* **2018**, *18*.
2. Jeyanthi Rebecca, L.; Sharmila, S.; Das, M.P.; Seshiah, C. Extraction and Purification of Carotenoids from Vegetables. *J Chem Pharm Res* **2014**, *6*.
3. Ali Shehat, W.; Sohail Akh, Md.; Alam, T. Extraction and Estimation of Anthocyanin Content and Antioxidant Activity of Some Common Fruits. *Trends Appl Sci Res* **2020**, *15*, doi:10.3923/tasr.2020.179.186.
4. Rahman, M.M.; Rahaman, M.S.; Islam, M.R.; Hossain, M.E.; Mithi, F.M.; Ahmed, M.; Saldías, M.; Akkol, E.K.; Sobarzo-Sánchez, E. Multifunctional Therapeutic Potential of Phytocomplexes and Natural Extracts for Antimicrobial Properties. *Antibiotics* 2021, *10*.
5. Ahmadi, A.; Shahidi, S.A.; Safari, R.; Motamedzadegan, A.; Ghorbani-HasanSaraei, A. Evaluation of Stability and Antibacterial Properties of Extracted Chlorophyll from Alfalfa (Medicago Sativa L.). *Food and Chemical Toxicology* **2022**, *163*, doi:10.1016/j.fct.2022.112980.
